# What’s in a name: The role of verbalization in reinforcement learning

**DOI:** 10.3758/s13423-024-02506-3

**Published:** 2024-05-20

**Authors:** Jessica V. Schaaf, Annie Johansson, Ingmar Visser, Hilde M. Huizenga

**Affiliations:** 1 Department of Psychology, University of Amsterdam, Amsterdam, The Netherlands; 2 Cognitive Neuroscience Department, Radboud University Medical Centre, Nijmegen, The Netherlands; 3 Donders Institute for Brain, Cognition and Behaviour, Nijmegen, The Netherlands; 4 Yield, Research Institute for Child Development and Education, Amsterdam, The Netherlands; 5 ABC, Amsterdam Brain and Cognition Centre, Amsterdam, The Netherlands

**Keywords:** Reinforcement learning, Verbalization, Cognitive processes, Interference

## Abstract

Abstract (e.g., characters or fractals) and concrete stimuli (e.g., pictures of everyday objects) are used interchangeably in the reinforcement-learning literature. Yet, it is unclear whether the same learning processes underlie learning from these different stimulus types. In two preregistered experiments (N = 50 each), we assessed whether abstract and concrete stimuli yield different reinforcement-learning performance and whether this difference can be explained by verbalization. We argued that concrete stimuli are easier to verbalize than abstract ones, and that people therefore can appeal to the phonological loop, a subcomponent of the working-memory system responsible for storing and rehearsing verbal information, while learning. To test whether this verbalization aids reinforcement-learning performance, we administered a reinforcement-learning task in which participants learned either abstract or concrete stimuli while verbalization was hindered or not. In the first experiment, results showed a more pronounced detrimental effect of hindered verbalization for concrete than abstract stimuli on response times, but not on accuracy. In the second experiment, in which we reduced the response window, results showed the differential effect of hindered verbalization between stimulus types on accuracy, not on response times. These results imply that verbalization aids learning for concrete, but not abstract, stimuli and therefore that different processes underlie learning from these types of stimuli. This emphasizes the importance of carefully considering stimulus types. We discuss these findings in light of generalizability and validity of reinforcement-learning research.

**Supplementary Information:**

The online version contains supplementary material available at 10.3758/s13423-024-02506-3.

## Introduction

In reinforcement-learning studies, people learn which stimulus yields the highest reward. These stimuli are usually abstract such as foreign language characters (e.g., Daw et al., 2011; Frank et al., 2004; Pessiglione et al., 2006) or fractals (e.g., Gläscher et al., 2010). To study reinforcement-

learning processes in developmental and aging populations, abstract stimuli are often replaced by concrete pictures of, for example, everyday objects (e.g., Eppinger et al., 2009; Eppinger & Kray, 2011; van de Vijver et al., 2015; van den Bos et al., 2009; Xia et al., 2021). This raises the question of whether the same learning processes underlie reinforcement learning of abstract and concrete stimuli. In the current preregistered study, we therefore tested whether abstract and concrete stimuli yield different reinforcement-learning performance, and whether potential differences are due to verbalization.

A recent reinforcement-learning study (Farashahi et al., 2020) showed superior learning for concrete as compared to abstract stimuli. However, the mechanism underlying this superior learning remains understudied. A potential mechanism is verbalization, that is, naming stimuli while learning, as it is hypothesized to modulate otherwise non-verbal cognitive processes (Kray et al., 2015; Lupyan, 2012). Specifically, verbalization may aid reinforcement-learning performance as it helps keep information in working memory. According to Baddeley’s classic working-memory model (1986; for a more recent review, see Baddeley & Hitch, 2019), a part of the working-memory system, called the phonological loop, temporarily stores verbal information and rehearses this information through inner speech. If stimuli are easier to encode phonologically, it is easier to appeal to this phonological loop while learning. We argue that concrete stimuli are easier to name than abstract ones, which makes them easier to encode phonologically, subsequently resulting in better reinforcement-learning performance for concrete than for abstract stimuli.

In support of this idea, there is ample evidence for a general role of working memory in reinforcement learning, that is, that people tend to rely on working memory (as opposed to associative reinforcement learning) when the number of things to learn fall within people’s working-memory capacity (e.g., Collins, 2018; Collins & Frank, 2012). In addition, experimental studies already showed that verbalization can aid performance in various (otherwise non-verbal) cognitive processes, including working memory (Forsberg et al., 2020; Souza & Skóra, 2017), category learning (Lupyan et al., 2007; Lupyan & Casasanto, 2015; Minda & Miles, 2010; Vanek et al., 2021; Waldron & Ashby, 2001; Zeithamova & Maddox, 2006; Zettersten & Lupyan, 2020), and motor learning (Gidley Larson & Suchy, 2015). Two recent studies (Radulescu et al., 2022; Yoo et al., 2023) addressed whether verbalization can also aid reinforcement learning. Yoo and colleagues (2023) showed that people performed worse in a condition in which concrete pictures represented the same object than in a condition in which the pictures represented different objects, concluding that verbal discriminability (i.e., distinguishable stimulus names) is particularly important for learning. Similarly, Radulescu and colleagues (2022) showed that people performed worse in a condition in which stimuli were difficult to verbalize than in a condition in which they were easy to verbalize (see also Waltz et al., 2007). Both studies drew conclusions about the effects of verbal processes on learning based on indirect measures of verbalization, that is, they relied on the assumption that verbalization was affected differently in different conditions. We implemented a direct measure of the effects of verbalization on learning. Specifically, we adopted a dual-task design in which people learned abstract and concrete stimuli while verbalization was either hindered or unhindered. Such a dual-task design allows one to assess whether a certain cognitive process plays a larger role in one condition than in another (Pashler, 1994). As we were specifically interested in whether verbalization plays a larger role when learning concrete stimuli than when learning abstract stimuli, we adopted a dual task that suppresses verbalization, that is, we let participants count to three. Doing this while learning, a procedure commonly applied when investigating the effects of verbalization (Nedergaard et al., 2023), suppresses participants’ ability to rehearse verbal information in their phonological loop (e.g., Baddeley & Larsen, 2007; Miyake et al., 2004), precluding them from using verbalization to aid learning.

## Experiment 1

We primarily hypothesized an interaction effect between stimulus type and verbalization condition on accuracy, that is, that the detrimental effect of hindered verbalization would be more pronounced for concrete compared to abstract stimuli. In addition, we expected main effects of both stimulus type and verbalization condition on accuracy. That is, better learning for concrete compared to abstract stimuli (Farashahi et al., 2020) and better learning in the unhindered verbalization condition because it only requires performing a single task (Pashler, 1994). We also expected to observe these effects in interaction with trial, that is, that hindered verbalization would especially lead to slower learning for concrete stimuli (stimulus type x verbalization condition x trial interaction), that learning would be faster for concrete than abstract stimuli (stimulus type x trial), and that learning would be faster in the absence compared to the presence of the verbalization task (verbalization condition x trial).

### Method

#### Preregistration

All procedures and analyses were preregistered within the Open Science Framework as *Reinforcement learning of abstract vs concrete stimuli* (https://osf.io/qwu3g). These analyses are labeled confirmatory analyses. Any other analyses are considered exploratory, and are specified as such. Data and analysis code are freely available at https://osf.io/w9fv4/.

#### Participants

A power analysis for the multilevel logistic regression on accuracy (Olvera Astivia et al., 2019) with medium effect sizes for the main effects (i.e., 0.5) and a small effect size for the interaction of interest (i.e., 0.25) indicated a required sample size of 50 to detect the crucial interaction between stimulus type and verbalization condition with a power of 0.9. As such, a total of 68 participants were recruited through the University of Amsterdam. A total of 18 participants were excluded, either because they did not perform the verbalization task correctly (either forgetting to count on more than six beats in a row or more than 25 beats in total, as checked by a present experimenter; *n* = 16) or because of technical failures (*n* = 2). We did not exclude any participants based on their learning performance because we anticipated that if hindered verbalization would affect learning in abstract stimuli, performance in this condition could drop to chance level. Thus, the final sample consisted of 50 participants (24 female, one other*, M*_*age*_ = 21.5 (3.0) years, range: 18–33 years). We only recruited participants without experience with the Hiragana alphabet or character-based languages to minimize individual differences in the ability to verbalize these abstract stimuli. We reimbursed participants when they completed the long-term retention task within 36 h after testing (see section *Reinforcement-learning task*) and, as preregistered, performed analyses on data from these participants (*n* = 44).

#### Reinforcement-learning task

##### Experimental design

We adopted a 2 x 2 within-subjects design in which we manipulated stimulus type (i.e., abstract vs. concrete) and verbalization condition (i.e., hindered vs. unhindered). Each participant performed one block of each combination (four blocks in total) in a randomized order with one constraint: only one of the manipulations changed between two subsequent blocks. For example, an abstract block with verbalization task was followed by either a concrete block with verbalization task (different stimulus type) or an abstract block without verbalization task (different verbalization condition). Each block was followed by a testing block (short-term retention). After 24–36 h participants again performed a testing block (long-term retention).

##### Task design

**Fig. 1:**
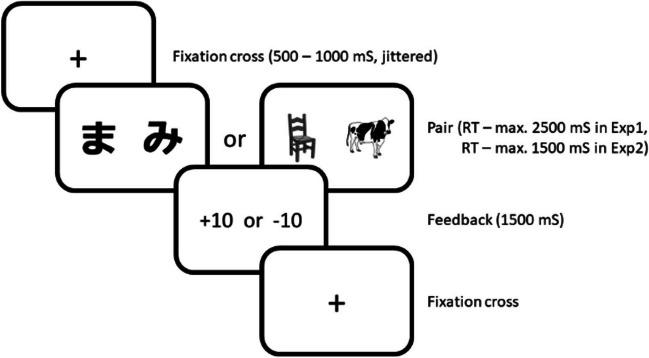
Example trial sequence of the reinforcement-learning task. *Note*. In the learning task, each trial started with a fixation cross for 500–1,000 ms, jittered in steps of 50 ms, to make the metronome beats and the timing of the stimulus pair uncorrelated. Hereafter a pair was presented from which the participant chose one within either 2,500 ms (Exp. 1) or 1,500 ms (Exp. 2). Once a participant chose, feedback was presented for 1,500 ms; hereafter a fixation cross signaled the next trial. If a participant failed to choose within the response window, “Too late! Respond faster!” appeared on the screen for 1,500 ms. These trial sequences were the same across the two experiments (except for the response window) and across the verbalization conditions. Timed-out responses were excluded from all analyses. In Experiment 1, the percentage of timed-out responses ranged between 0% and 3.1% across participants with 0.6% on average. In Experiment 2, it ranged between 0.4% and 10.2% across participants with 3.9% on average. In both experiments, including these timed-out responses as incorrect responses did not alter the pattern of results; significance only changed for a secondary interaction (i.e., between verbalization condition and trial) in Experiment 2 (see OSM Table II)

As illustrated in Fig. 1, in each learning block, we presented participants with four new stimulus pairs from which they learned the stimulus with the highest expected value, that is, the correct stimulus, based on feedback. Specifically, we instructed participants that their goal was “to win as many points as possible by clicking on one of the stimuli” and told them that the more points they earned, the higher the monetary bonus they would receive. The stimuli in a pair were fixed across the trials of a block and each pair was presented 16 times (64 trials per block, four blocks, resulting in a fixed total of 256 trials for all participants). The order of the pairs was randomized per four trials such that pairs were presented a maximum of twice in a row. In two of the four blocks (i.e., the unhindered verbalization blocks), participants heard a metronome (80 bpm) but did not have to say anything. In the other two blocks (i.e., the hindered verbalization blocks), we instructed participants to say “1, 2, 3” repeatedly out loud on the beat of the metronome during learning. This articulatory-suppression manipulation hinders verbalization by occupying the phonological loop (Baddeley et al., 1984; Emerson & Miyake, 2003).

Immediately after each learning block, a testing block followed. In this testing block, the same pairs as in the learning block were presented, four times each (randomized per four trials). Participants were asked to indicate the correct stimulus (formulated as: “Please click on the stimulus you think usually resulted in winning 10 points”), but did not receive any feedback on their choice. As such, performance in testing blocks did not count towards their reimbursement. The testing block was self-paced (i.e., without response deadline). After 24–36 h, participants again performed a testing block, but now with all 16 pairs (each presented four times, randomized per 16 trials).

##### Practice

Before each learning block, participants practiced the new stimulus type and verbalization condition combination. In this practice block, two stimulus pairs were presented for eight trials each (totaling to 16 trials).

##### Stimuli

We selected stimuli that are commonly used in reinforcement-learning studies because we aimed to uncover the potential role of verbalization in such studies. As abstract stimuli, we used characters from the Hiragana alphabet (for examples, see Frank et al., 2004; Hämmerer et al., 2011; Simon et al., 2010). As concrete stimuli we used pictures of everyday objects^1^ (for examples, see Eppinger et al., 2009; Eppinger & Kray, 2011; van de Vijver et al., 2015; van den Bos et al., 2009; Xia et al., 2021) from the MultiPic database (Duñabeitia et al., 2018); we only considered stimuli with average visual complexity and one-syllable names in both English and Dutch. To only select stimuli with similar verbalizability and to assess whether the abstract stimuli were indeed harder to verbalize than the concrete ones, we conducted a pilot study in which we asked participants to come up with a name for the stimuli and to indicate how difficult it was to do so. To select stimuli that were similarly difficult to discriminate, we also asked participants to rate how similar they found the stimuli in a pair. For details on this stimulus selection procedure and pilot results, we refer to Online Supplemental Material (OSM) Text I.

##### Feedback

In all learning blocks, choices for one, which we coin the correct, stimulus would usually lead to winning 10 points (75% of trials), and only sometimes to losing 10 points (25% of trials). Choices for the other, incorrect, stimulus would usually lead to losing 10 points (75% of trials), and only sometimes to winning 10 points (25% of trials). Which stimulus was correct was determined randomly per participant.

##### Procedure

Participants were tested individually in a lab cubicle. They sat in front of a laptop with mouse and keyboard and received on-screen instructions about the learning and short-term retention tasks. An experimenter was always present during testing to check whether the participant performed the verbalization task (i.e., saying “1, 2, 3” on the beat of the metronome) correctly. This on-site experiment took approximately 30 min. At the end of the on-site experiment, participants saw the bonus they earned on the screen, were asked how they experienced the experiment (both on-screen and by the experimenter), and were informed about the long-term retention task. After 24 h, participants received a link to this task via email. They were instructed to complete it at home within 12 h (i.e., 24–36 h after the learning task). If participants did not complete the long-term retention task in time, they received €5 or course credits as reimbursement. If participants did complete the task in time, they received the reimbursement plus a bonus equal to the number of points won in the learning task divided by the number of trials (i.e., 256). This resulted in a bonus between €0 and €10 (*M*_*bonus*_ = €1.39 (€0.95)). We told participants that winning more points would lead to a higher reimbursement, but they were unaware of the formula used to convert points into money. After completing the learning task, they were informed that bonus money would only be paid out when the long-term retention task was completed in time.

#### Data analyses

##### Learning

To test whether abstract and concrete stimuli yield different reinforcement-learning performance and whether potential differences are due to verbalization, we performed a multilevel logistic regression analysis on trial-by-trial choice accuracy in the learning blocks of the reinforcement-learning task; we did so using the glmer function from the lme4 package (Bates et al., 2015). We modeled fixed effects of stimulus type (abstract (coded as -1) versus concrete (coded as 1)), verbalization condition (hindered (coded as -1) versus unhindered (coded as 1)), trial (linear, centered, such that all effects excluding trial are estimated in the middle of learning), and all two- and three-way interactions, as well as random intercepts and random slopes for the main effects. We fixed covariances between random effects to zero.

##### Retention

To test for these same effects on retention rates, we performed a multilevel linear regression analysis on short- and long-term retention rates, defined as the average proportion correct in the testing blocks, that is, collapsed across pairs and trials; we did so using the lmer function from the lme4 package (Bates et al., 2015). We modeled fixed effects of stimulus type, verbalization condition, delay (short vs. long) and all two- and three-way interactions, as well as random intercepts and random slopes for the main effects. Covariances between random effects were fixed to zero.

##### Response times

Finally, we performed an exploratory multilevel linear regression analysis on trial-by-trial response times (irrespective of choice accuracy). We modeled fixed effects of stimulus type, verbalization condition, trial, and all two- and three-way interactions, random intercepts and random slopes for the main effects, and fixed covariances between random effects to zero. Also, we modeled first-order autoregression to take the autocorrelation between the error term across trials into account. We did this using the lme function from the nlme package (Pinheiro et al., 2022).

### Results

#### Confirmatory analyses: Learning and retention

##### Learning

**Fig. 2:**
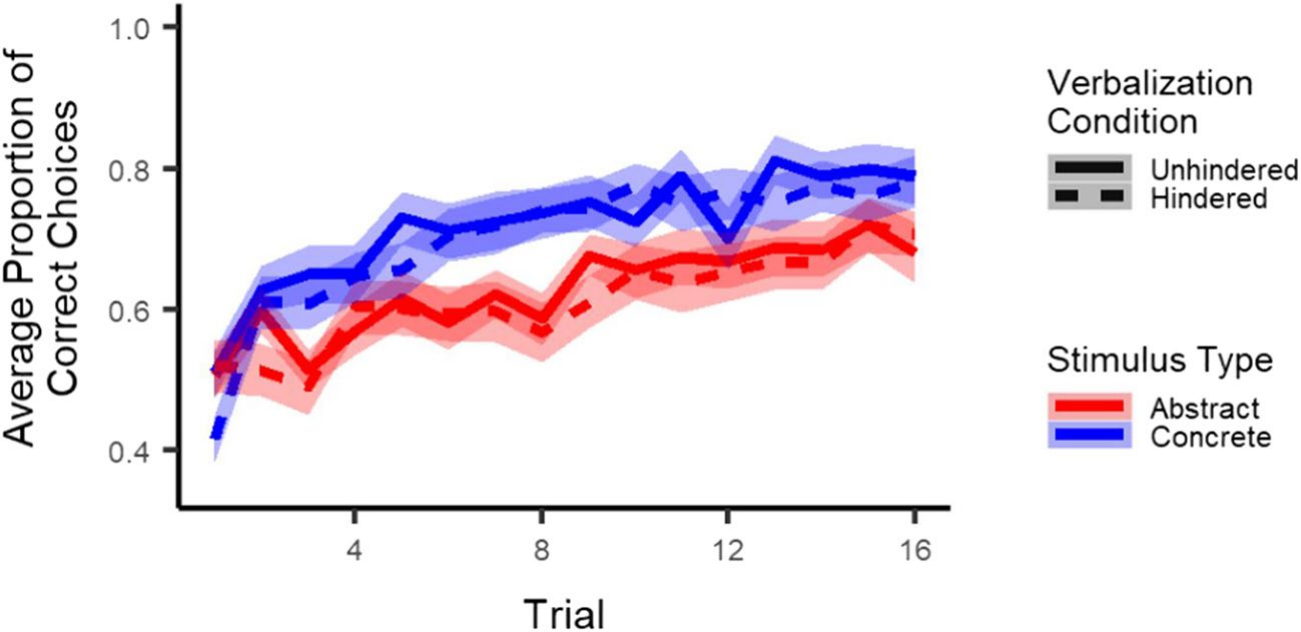
Learning: Participants learned to choose the correct stimulus across trials and did this better for concrete than abstract stimuli. Hindered verbalization did not affect learning for either abstract or concrete stimuli. *Note*. The shaded area corresponds to one standard error of the mean. The x-axis represents trials collapsed across the four pairs presented in each block. Results from exploratory multilevel logistic regression analyses on accuracy in all four conditions separately indicated that participants learned in all conditions, with linear trial estimates ranging from 1.2 to 2.2 and all *p*s < .001

Most importantly, as illustrated in Fig. 2, results showed no interaction between stimulus type and verbalization condition (*p* = .51) and no three-way interaction between stimulus type, verbalization condition, and trial (*p* = .76). Thus, in contrast to our expectations, the effect of the verbalization task did not differ for abstract and concrete stimuli. Results did show a main effect of stimulus type (*z* = 4.03, *p* < .001), indicating higher accuracy for concrete than abstract stimuli, and an interaction between stimulus type and trial (*z* = 3.2, *p* = .001), indicating accuracy improved faster across trials for concrete as compared to abstract stimuli. Finally, results showed neither a main effect of verbalization condition (*p* = .27) nor an interaction between verbalization condition and trial (*p* = .52).

##### Retention

**Fig. 3:**
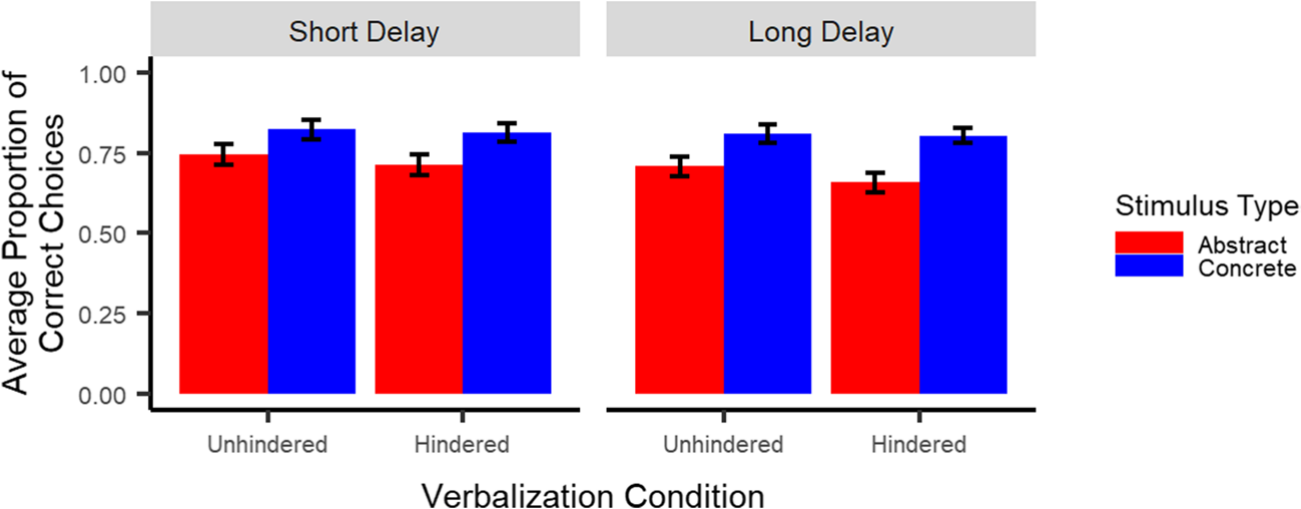
Retention: Participants recalled the correct stimulus better for concrete than abstract stimuli, but not better in the unhindered as compared to the hindered condition. *Note*. Error bars represent one standard error of the mean. To obtain the proportion of correct choices, choice accuracy was averaged across pairs and trials

Most importantly, as illustrated in Fig. 3, results showed neither a stimulus type x verbalization condition interaction (*p* = .21), indicating no difference in the effect of the verbalization task across stimulus types, nor a stimulus type x verbalization condition x delay interaction (*p* = .74), indicating this effect did not differ between short and long delay. Results did show a main effect of stimulus type (*t*(48.1) = 4.6, *p* < .001), indicating better retention for concrete than abstract stimuli, but no stimulus type x delay interaction (*p* = .11). In addition, results showed neither a main effect of verbalization condition (*p* = .31), nor a verbalization condition x delay interaction (*p* = .86). Finally, they did show slightly better retention after short than long delay (main effect of delay: *t*(228) = -2.0, *p* < .05).

Taken together, these results suggest no differential effect of the verbalization task on learning from, and retention of, abstract versus concrete stimuli. They show in addition that both learning and retention were better for concrete as compared to abstract stimuli, and that learning and retention were unaffected by the verbalization task.

#### Exploratory analyses: Response times

**Fig. 4:**
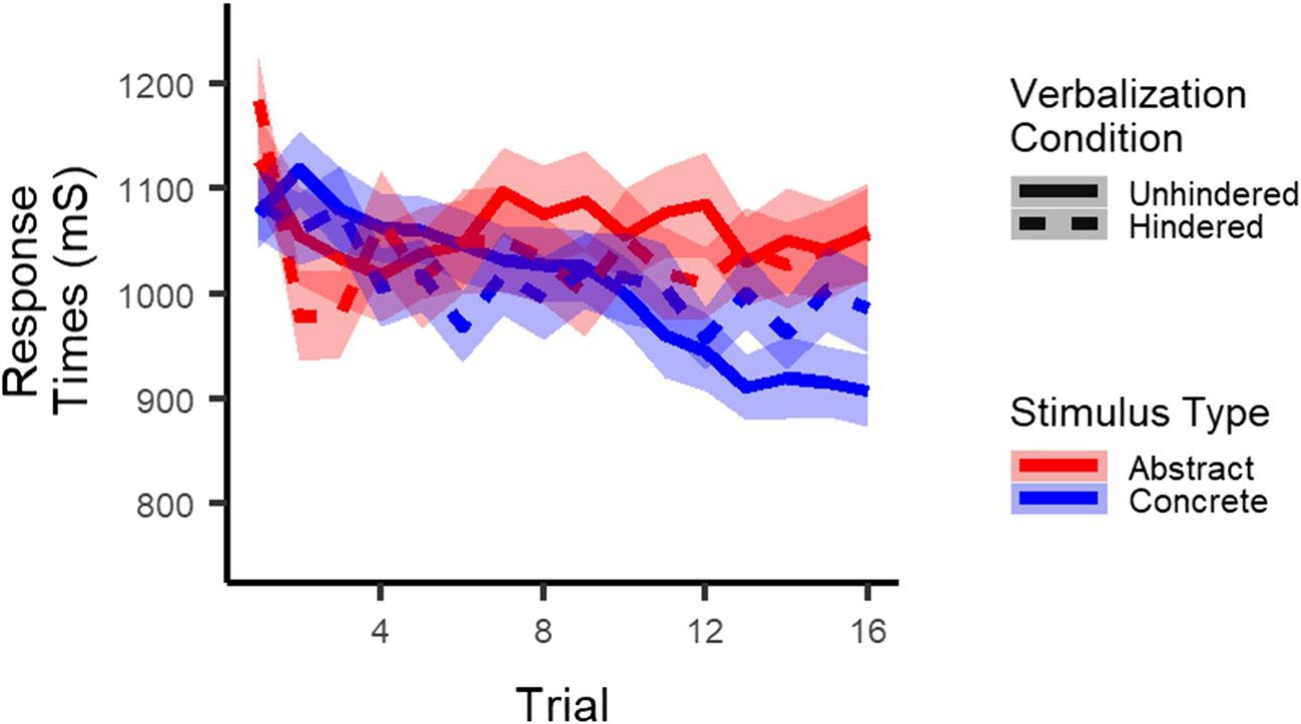
Response times: Participants responded slower to concrete stimuli when verbalization was hindered than when it was not. This was not the case for abstract stimuli. *Note*. The shaded area corresponds to one standard error of the mean. The x-axis represents trials collapsed across the four pairs presented in each block

One possible explanation for the lack of effect of the verbalization task on accuracy is that participants slowed down in order to keep up their performance, that is, that participants traded off their speed and accuracy (e.g., Wickelgren, 1977). We therefore performed an exploratory multilevel regression analysis on response times. Most importantly, as displayed in Fig. 4, results showed an interaction between stimulus type and verbalization condition (*t*(12743) = -2.3, *p* = .02) and a three-way interaction between stimulus type, verbalization condition, and trial (*t*(12743) = -3.4, *p* = .001). Follow-up analyses in each stimulus type separately only indicated a detrimental effect of hindered verbalization on response times for concrete stimuli (main effect of verbalization condition: *p* = .77; verbalization x trial interaction: *t*(6347) = -4.8, *p* < .001), not for abstract ones (both *p*s > .50). In addition, results showed no main effect of stimulus type (*p* = 0.10), but did show an interaction between stimulus type and trial (*t*(12743) = -6.3, *p* < .001), indicating that response times decreased faster for concrete as compared to abstract stimuli. Finally, results did not show a main effect of verbalization condition (*p* = .73), but did show an interaction between verbalization condition and trial (*t*(12743) = -2.8, *p* = .006), indicating that response times decreased faster in the unhindered than the hindered verbalization condition.

### Interim conclusion

We predicted that the detrimental effect of hindered verbalization on learning and retention would be more pronounced for concrete than for abstract stimuli. However, results did not show any effects of the verbalization task on learning or retention. Rather, exploratory analyses revealed our predicted interactions between stimulus type and the verbalization task on response times.

## Experiment 2

The result that we found our predicted interactions on response times instead of on accuracy may be explained by a speed-accuracy trade-off. To test this explanation, we performed a second experiment in which we prevented participants from slowing down by reducing the response window (as commonly done in the response-time literature; Wickelgren, 1977) and investigated stimulus type and hindered verbalization effects on accuracy, retention, and response times. We followed the same preregistered procedure as in Experiment 1 with three changes: we reduced the response window in the learning task from 2.5 to 1.5 s, we added a practice retention block, and performed the exploratory analysis on response times in a confirmatory manner (see section *Reinforcement-learning task and data analyses*).

With such a short response window, we expected the results found in Experiment 1 on response times to now become apparent on accuracy. Thus, with respect to accuracy, we predicted a detrimental effect of hindered verbalization on learning specifically in concrete stimuli. With respect to response times, we predicted no differential effect of hindered verbalization on abstract and concrete stimuli.

### Method

#### Participants

We recruited 58 participants who did not participate in Experiment 1 through the University of Amsterdam using the same exclusion criteria as in Experiment 1. Data from eight participants were excluded because they failed to perform the verbalization task correctly (*n* = 7) or because of technical failures (*n* = 1). The final sample thus consisted of 50 participants (26 female, *M*_*age*_ = 21.1 (2.4) years, range = 18–30 years). Participants received €5 or research credits as reimbursement plus a variable bonus between €0 and €10 (*M*_*bonus*_ = €1.21 (€1.06)). We only paid participants their earned bonus when they completed the long-term retention task within 36 h after testing and, as preregistered, performed analyses on the long-term retention data from this smaller sample

(*n* = 42).

#### Reinforcement-learning task and data analyses

We administered the same reinforcement-learning task as described in Experiment 1 (see section *Reinforcement-learning task*), but reduced the response window from 2.5 s to 1.5 s and added a practice short-term retention block. We did the former to prevent participants from slowing down to keep up their performance, potentially explaining the absence of verbalization effects on accuracy in Experiment 1. We did the latter to familiarize participants with the task design before learning in the first block. We then performed confirmatory multilevel regression analyses on accuracy, short- and long-term retention rates, and response times (see section *Data analyses*).

### Results

#### Confirmatory analyses: Learning, retention, and response times

##### Learning

**Fig. 5:**
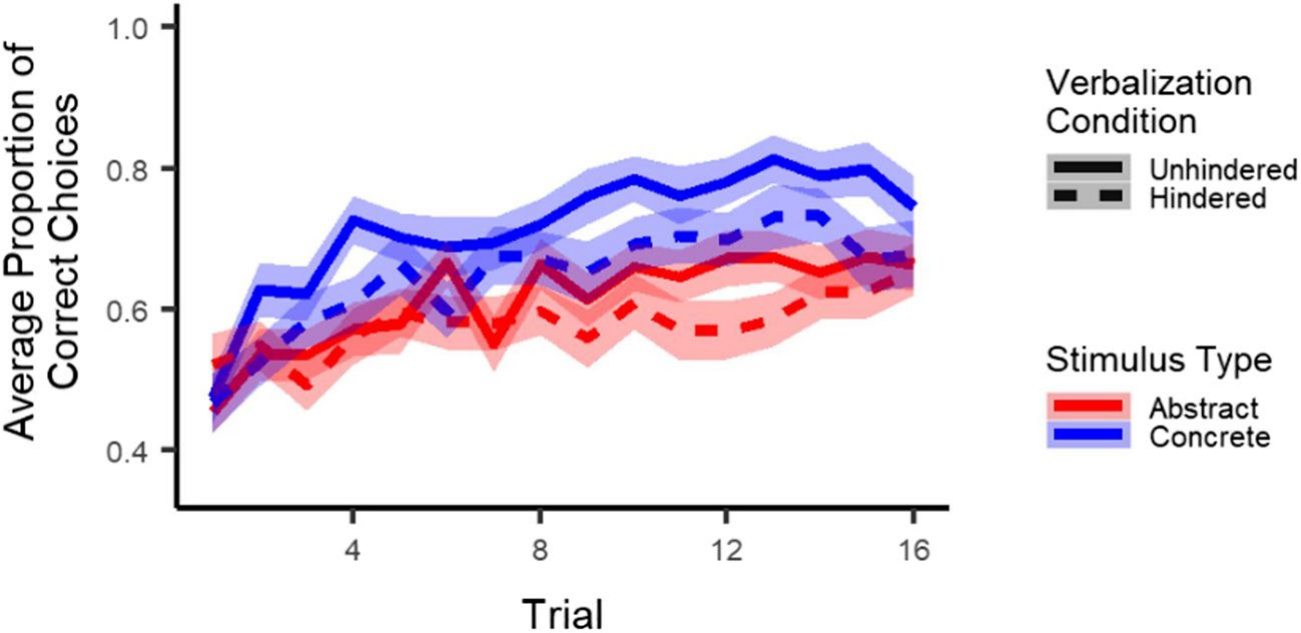
Learning: When learning concrete stimuli, participants chose the correct stimulus less often when verbalization was hindered than when it was unhindered. When learning abstract stimuli, this was not the case. *Note*. The shaded area corresponds to one standard error of the mean. The x-axis represents trials collapsed across the four pairs presented in each block. Results from exploratory multilevel logistic regression analyses on accuracy in all four conditions separately indicated that participants learned in all conditions, with linear trial estimates ranging from 0.5 to 1.9 and all *p*s < .003

Most importantly, as illustrated in Fig. 5, results showed a stimulus type x verbalization condition interaction (*z* = 3.1*, p* = .002), but no three-way interaction between stimulus type, verbalization condition, and trial (*p* = .98). Follow-up analyses in each stimulus type separately showed that the verbalization task only lowered accuracy in concrete stimuli (main effect of verbalization condition: *z* = 3.1, *p* = .002), not in abstract ones (*p* = .12). Thus, in accordance with our hypothesis, results showed that hindering verbalization specifically affected accuracy in concrete stimuli. In addition, results showed a main effect of stimulus type (*z* = 4.6, *p* < .001) and an interaction between stimulus type and trial (*z* = 4.4, *p* < .001), indicating higher accuracy and a faster improvement across trials for concrete than for abstract stimuli. Results also showed a main effect of verbalization condition (*z* = 3.0, *p* = .003), indicating higher accuracy in the absence than presence of the verbalization task, and a verbalization condition x trial interaction (*z* = 2.9, *p* = .004), indicating accuracy improved faster in the absence of the verbalization task as compared to in its presence.

##### Retention

**Fig. 6:**
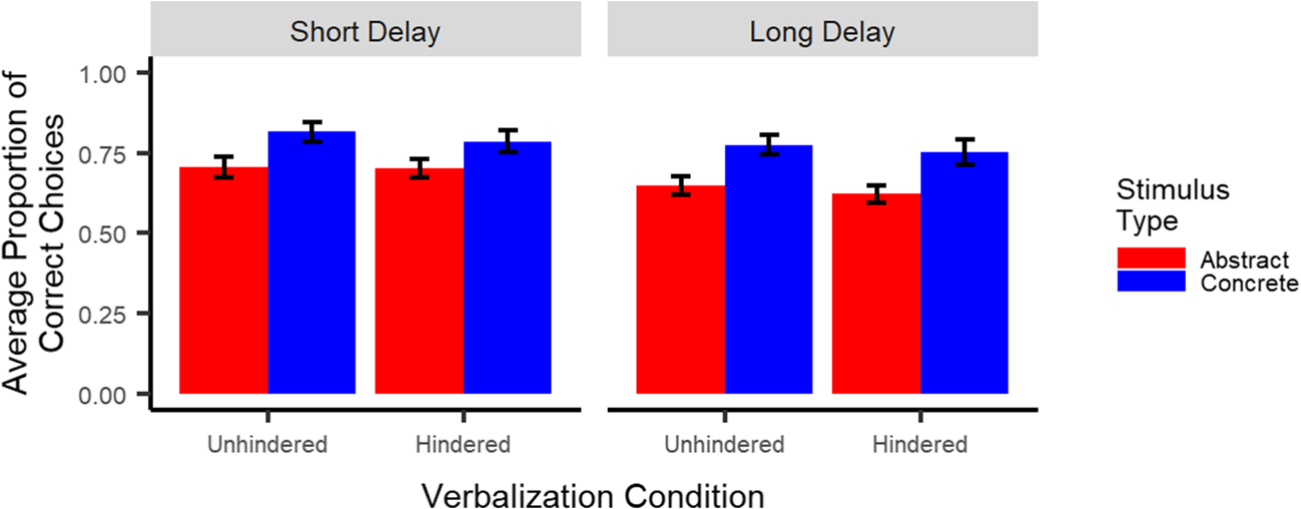
Retention: Participants recalled the correct stimulus better for concrete than abstract stimuli, but not better in the unhindered as compared to the hindered condition. *Note*. Error bars represent one standard error of the mean. To obtain the proportion of correct choices, choice accuracy was averaged across pairs and trials

As illustrated in Fig. 6, results showed no effects including verbalization condition (all *p*s > .32), indicating hindered verbalization did not interfere with retention for either abstract or concrete stimuli. Results did show a main effect of stimulus type (*t*(51) = 3.6, *p* = .001), indicating better retention for concrete than abstract stimuli, but no stimulus type x delay interaction (*p* = .91). Finally, they showed better retention after short than long delay (*t*(226) = -3.8, *p* < .001).

##### Response times

**Fig. 7:**
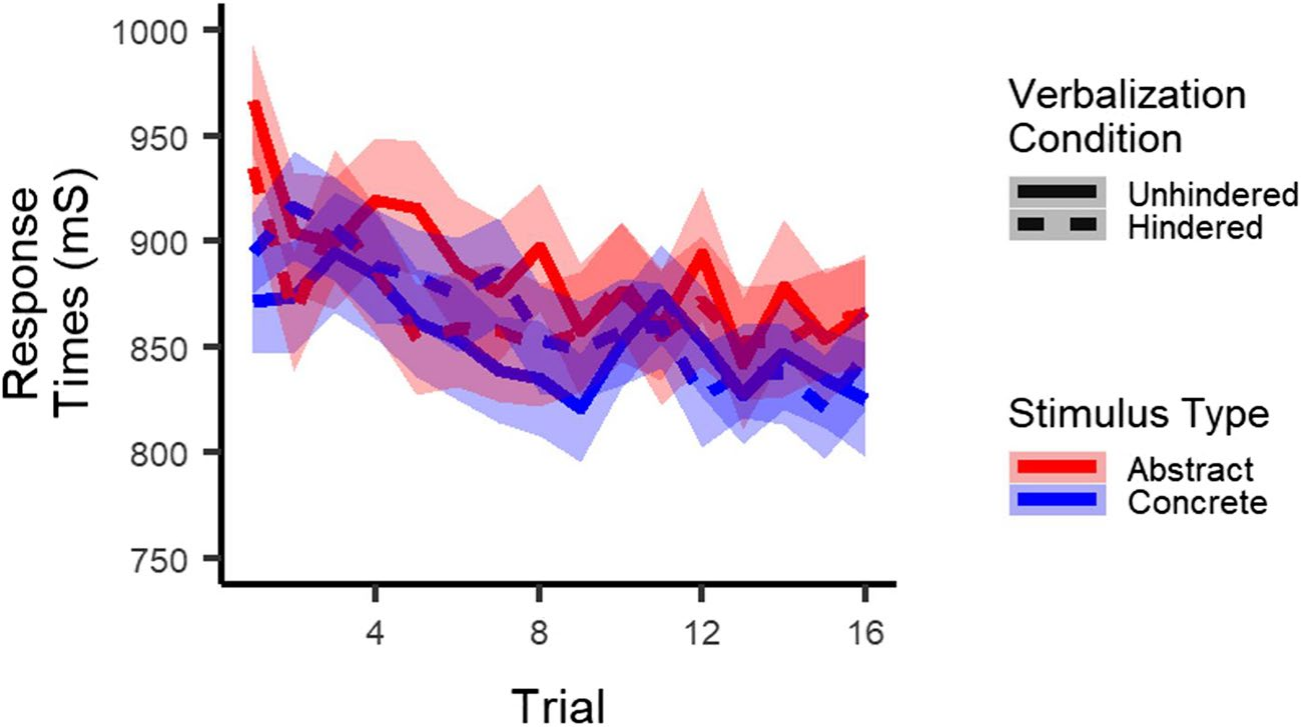
Response times: Participants responded similarly fast, irrespective of whether they learned abstract or concrete stimuli and of whether or not verbalization was hindered. *Note*. The shaded area corresponds to one standard error of the mean. The x-axis represents trials collapsed across the four pairs presented in each block

As displayed in Fig. 7, response-time results showed a stimulus type x verbalization condition interaction (*t*(12743) = -3.2, *p* = .001) and a three-way interaction between stimulus type, verbalization condition, and trial (*t*(12743) = 2.4, *p* = .02). However, follow-up analyses in each stimulus type separately indicated that all verbalization condition and verbalization condition x trial effects were non-significant (all *p*s > .07). In addition, results showed neither a main effect of stimulus type (*p* = .31) nor a stimulus type x trial interaction (*p* = .83), and neither a main effect of verbalization condition (*p* = .85) nor a verbalization condition x trial interaction (*p* = .92).

## Interim conclusion

Results from Experiment 2, in which we reduced the response window, showed the predicted detrimental effect of hindered verbalization on accuracy for concrete stimuli, but not abstract ones. In addition, they did not show this effect on response times.

## General discussion

In this preregistered study, we assessed whether abstract and concrete stimuli yield different reinforcement-learning performance, and whether potential differences are due to verbalization. To do so, we administered a reinforcement-learning task in which participants learned either type of stimuli while we hindered verbalization or not. Most importantly, our results showed that hindering verbalization interfered more with learning concrete than with learning abstract stimuli, as reflected in response times in Experiment 1, in which the response window was long, and in choice accuracy in Experiment 2, in which the response window was short. The results thus suggest that people rehearse stimulus names while learning stimuli that are easy to verbalize, which in turn aids their learning.

Our main result, that is, a more pronounced detrimental effect of hindered verbalization on concrete than abstract stimuli, corroborates recent studies suggesting that learning is difficult when stimuli are verbally difficult to discriminate (Yoo et al., 2023) and that stimuli that are easy to verbalize are easier to learn than difficult-to-verbalize ones (Radulescu et al., 2022). We extend these findings by directly showing that verbalization underlies this superior learning for verbalizable stimuli and by showing that this effect is specific to learning, not to retention. As our data showed that participants learned in all conditions (i.e., when verbalization was both hindered and unhindered, and for both abstract and concrete stimuli) and that hindered verbalization only affects learning for concrete stimuli, this implies that only reinforcement learning underlies learning abstract stimuli, whereas both reinforcement learning and verbalization underlie learning concrete stimuli. Our main result thus provides additional evidence that working-memory processes are involved in reinforcement-learning tasks (Collins & Frank, 2012, 2018; Yoo & Collins, 2022), at least in concrete stimuli.

This finding has far-reaching implications for the reinforcement-learning field and related fields that use various stimulus types. Specifically, it suggests that studies using different types of stimuli may be incomparable, affecting the generalizability of results. For instance, brain processes associated with learning abstract stimuli (e.g., Daw et al., 2011; Frank et al., 2005; Palminteri et al., 2015; Pessiglione et al., 2006) may not be generalizable to such processes associated with concrete stimuli (e.g., Eppinger et al., 2008; van den Bos et al., 2009). Also, this finding suggests that stimulus choice affects the validity of results. For instance, when a study using concrete stimuli finds developmental effects on learning, it is unclear which development it measures: the development of reinforcement-based learning, or the development of verbalization, an ability shown to increase from childhood to young adulthood (Yeates, 1994) and to decrease again in older adulthood (Au et al., 1995; Zec et al., 2007). We therefore believe it valuable to assess the comparability of existing studies by performing meta-analyses in which stimulus type is included as fixed or random effect (Yarkoni, 2022).

Our main questions pertained to the differential role of verbalization in learning different types of stimuli. Other results are worth discussing as well. In line with previous studies, we found that learning was easier for concrete than abstract stimuli (Farashahi et al., 2020). Using our dual-task design, we showed that concrete stimuli are easier to verbalize and therefore easier to learn. However, there may be coexisting explanations of this stimulus-type effect. First, results from a pilot study showed that the abstract stimuli were less discriminable than the concrete ones (see OSM Text I). It could thus be that the concrete stimuli were easier to learn because the two concrete stimuli in a pair were visually more discriminable than the two abstract ones, as previously shown to affect learning (Schutte et al., 2017). Second, we used everyday objects as concrete stimuli while we excluded participants that were familiar with the Hirigana alphabet (our abstract stimuli) prior to testing. As such, superior learning for concrete stimuli could be explained by higher familiarity (e.g., Epstein et al., 1960; Stern et al., 2001). It is beyond the scope of the paper to further investigate which reason applies because we purposely stuck to stimuli commonly used in the literature and because we were mainly interested in the processes underlying learning from abstract and concrete stimuli, that is, in the interaction between stimulus type and the verbalization task. However, future studies could try to replicate our results using different designs. For instance, by replacing the characters by more discriminable fractals (see, e.g., Gläscher et al., 2010) or by comparing hindered verbalization effects between abstract and concrete stimuli after familiarizing participants with the stimuli (see, e.g., Radulescu et al., 2022).

We also found that participants learned better when they were not required to perform the verbalization task while learning. This is in line with a large literature on dual-task interference, suggesting that people have a hard time performing two tasks concurrently (e.g., Pashler, 1994).

Although non-significantly, our results suggested that the verbalization task not only interfered with learning concrete stimuli, but also with learning abstract ones. It is unclear whether this is because of general dual-task interference or because people specifically tend to verbalize abstract stimuli. Judging from participants’ comments after they completed the experiment, it seems like the latter explanation holds: participants indicated they tried to verbalize the abstract stimuli, but were less able to do so as compared to the concrete ones, making it harder to learn them. To experimentally test this, future studies could add a condition in which participants perform a concurrent task that does not involve verbalization – for example, a foot-tapping task (e.g., Emerson & Miyake, 2003) ﻿– and compare task effects across the three tasks. If people learn worse from abstract stimuli because they concurrently perform a second task, one would expect interference from both the verbalization and the foot-tapping task. If people learn worse from abstract stimuli because they cannot use verbalization to aid learning, one would only expect interference from the verbalization task.

Relatedly, because we administered one type of dual task, one may argue that the observed dual task interference was due to a process other than verbalization. First, one could argue that interference was merely due to general task interference. This account, however, does not explain our result that the dual task interfered more with learning concrete than abstract stimuli. Second, one could argue that interference was due to the dual task taking up general working-memory capacity instead of verbalization per se. However, as we used the same number of stimuli in both abstract and concrete conditions, this account also does not explain why the dual task interfered more with learning concrete than abstract stimuli. Third, one could argue that the concrete stimuli were visually more complex than the abstract ones and that interference was due to the dual task taking up visual instead of verbal working memory. However, as the dual task did not involve visual information, it is very unlikely that it interfered with visual storage. Fourth, one could argue that the dual task interfered with learning through long-term memory. That is, it could be that learning concrete stimuli requires learners to retrieve information from long-term memory more so than learning abstract stimuli, and that the dual task interferes with this process. As we used everyday objects as concrete stimuli, it could indeed be that learners take advantage of the familiarity of these objects and thus appeal to their long-term memory during learning. This would, however, not affect learning as this process doesn’t help them retrieve which stimulus is the correct one and can thus also not explain our main result.

Results from a pilot study, in which we assessed the verbalizability of the considered abstract and concrete stimuli, showed that, in general, concrete stimuli were easier to verbalize than abstract ones (see OSM Fig. II). Yet, exploratory analyses did not indicate effects of the degree of verbalizability on learning (see OSM Text II). It may be that this was because the stimuli within each stimulus type had similar degrees of verbalizability. To further investigate the effect of verbalization on learning, it may be worthwhile to investigate the effect of the degree of verbalizability in concrete stimuli. For instance, by administering a set of concrete stimuli with differing degrees of verbalizability. It may be that the effects of verbalization increase with the verbalizability of the concrete stimuli. However, it may also be that verbalizability only aids learning to a certain degree.

Finally, to investigate whether the same processes underlie learning from abstract and concrete stimuli, we adopted a dual-task design in which we hindered verbalization. Specifically, we occupied participants’ phonological loop (by letting them count while playing), making it more difficult for them to use verbalization to aid learning. In future studies, one may apply computational modeling to assess the origins of the differential effect of hindered verbalization on learning from the two types of stimuli. Ideally, one would want to disentangle the different components of the working-memory system by formulating computational models that separate general working-memory processes from verbal and visual processes. This would allow one to draw conclusions about the specific contributions of the different working-memory systems to learning without administering a concurrent verbalization task. However, to our knowledge, only models assessing general working-memory processes (e.g., Collins & Frank, 2012) have been developed for reinforcement-learning data, not models specifically explaining verbal working-memory processes.

## Conclusion

To conclude, our results suggest that learning concrete stimuli involves verbalization in addition to basic reinforcement learning. These findings emphasize the importance of carefully considering which stimuli to use in order to ensure the generalizability of results and to validly answer research questions.

## Supplementary Information

Below are the link to the electronic supplementary material.

Supplementary file1 (DOCX 22.7 KB)

Supplementary file2 (DOCX 21.8 KB)

Supplementary file3 (DOCX 22.4 KB)

Supplementary file4 (DOCX 23.2 KB)

Supplementary file5 (DOCX 21.7 KB)

Supplementary file6 (DOCX 140 KB)

Supplementary file7 (DOCX 136 KB)

Supplementary file8 (DOCX 55.3 KB)

## Data Availability

The datasets generated and analyzed during the current study are available via the Open Science Framework repository at https://osf.io/w9fv4/.

## Footnotes

1 Note that in commonly-used stimulus sets in the reinforcement-learning literature abstract/concrete is confounded with verbalizability (i.e., abstract stimuli are usually less verbalizable than concrete ones). This, however, does not take away from our main message: people may verbalize stimuli to aid learning, which is easier for verbalizable stimuli.

## Code availability

The analysis code is available via the Open Science Framework repository at https://osf.io/w9fv4/.
