# Supplementary material for: What’s in a name: The role of verbalization in reinforcement learning
Source: Psychon Bull Rev. 2024 May 20;31(6):2746–57. doi: 10.3758/s13423-024-02506-3 (PMC11680654; doi:10.3758/s13423-024-02506-3)
Supplement: Supplementary file 1 — Supplementary file1 (DOCX 22.7 KB) [file 13423_2024_2506_MOESM1_ESM.docx]

**Supplemental Text I: Stimulus selection procedure (verbalizability and similarity)**

We performed a pilot study to assess the verbalizability and similarity of the stimuli. To do so, as abstract stimuli, we first selected eligible characters from the Hirigana alphabet. We removed characters that were readily verbalizable such as those resembling numbers or letters from the alphabet. For the same reason, we removed characters that contained obvious shapes such as circles and squares. This left us with 28 characters which we divided into 14 pairs, making sure the two characters in a pair had the most distinguishable shapes as possible.

As concrete stimuli, we selected eligible pictures from the MultiPic database (Duñabeitia et al., 2018) based on the following criteria: (1) average visual complexity (i.e., between the 25^th^ and 75^th^ percentile in both Dutch and English; roughly between 2 and 3.5 on a 5-point scale), (2) similar visual complexity in English and Dutch (i.e., difference < 0.2), (3) single-syllable labels in English, (4) labels with less than 6 characters (to remove multiple-syllable labels in Dutch), and (5) labels with same number of characters in both English and Dutch. From the 65 pictures that met these criteria, the authors selected a subset of 16 pictures that were clear illustrations of everyday objects and animals and paired these such that the stimuli in a pair had different shapes and belonged to different categories.

Thereafter, we showed the 22 stimulus pairs (14 abstract and 8 concrete ones) to 14 participants and asked them to come up with a name for each stimulus (open question), timed how long they took to do this, asked how difficult they found this (on a scale from 1 “very easy” to 7 “very difficult”), and asked them to indicate how similar they found the stimuli in a pair (on a scale from 1 “very different” to 7 “very similar”).

As displayed in Supplemental Figure I, results showed that participants took longer to name abstract than concrete stimuli. Results also indicated that naming of one of the concrete stimuli took surprisingly long. Finally, results showed that participants found it more difficult to come up with a name for the abstract than for the concrete stimuli (Supplemental Figure II) and that they found the stimuli in abstract pairs more similar than in the concrete pairs (Supplemental Figure III).

These results led us to select 8 of the 14 abstract pairs to use in the experiment. We removed 6 abstract pairs because at least one of the stimuli in the pair was verbalized (as defined by at least three participants giving specific names). Moreover, the results led us to replace two concrete stimuli, both because they were difficult to name as reflected in inconsistent naming, slow response times and self-reported difficulty.

The 16 pairs used in the experiment can be found on https://osf.io/w9fv4/.

References

Duñabeitia, J. A., Crepaldi, D., Meyer, A. S., New, B., Pliatsikas, C., Smolka, E., & Brysbaert, M. (2018). MultiPic: A standardized set of 750 drawings with norms for six European languages. *Quarterly Journal of Experimental Psychology*, *71*(4), 808–816. https://doi.org/10.1080/17470218.2017.1310261
