# Supplementary material for: What’s in a name: The role of verbalization in reinforcement learning
Source: Psychon Bull Rev. 2024 May 20;31(6):2746–57. doi: 10.3758/s13423-024-02506-3 (PMC11680654; doi:10.3758/s13423-024-02506-3)
Supplement: Supplementary file 2 — Supplementary file2 (DOCX 21.8 KB) [file 13423_2024_2506_MOESM2_ESM.docx]

**Supplemental Text II: Multilevel regression analyses on accuracy including naming difficulty**

In a pilot study, intended to select suitable abstract and concrete stimuli, we asked participants how difficult they found it to come up with a name for the stimuli. We did this because difficulty in naming may lead to lower accuracy. To test this idea, we added naming difficulty (standardized within each stimulus type to obtain relative naming difficulty and averaged across the two stimuli in a pair) as obtained in the pilot study (note that these were different participants) as main effect to the multilevel regression analysis on accuracy. Results showed no relationship between naming difficulty and accuracy (*p* = .05 in Exp 1; *p* = .70 in Exp 2). With respect to the other effects, adding this regressor only slightly altered the results. In Exp 1, the interaction between stimulus type and verbalization condition became significant (*z* = 2.4, *p* = .02), indicating a stronger effect of the verbalization task for concrete than abstract stimuli (although the main effect of verbalization condition was non-significant for both stimulus types). In Exp 2, no effects changed in significance. These results thus suggest a minimal role of naming difficulty on accuracy.
