# Supplementary material for: What’s in a name: The role of verbalization in reinforcement learning
Source: Psychon Bull Rev. 2024 May 20;31(6):2746–57. doi: 10.3758/s13423-024-02506-3 (PMC11680654; doi:10.3758/s13423-024-02506-3)
Supplement: Supplementary file 3 — Supplementary file3 (DOCX 22.4 KB) [file 13423_2024_2506_MOESM3_ESM.docx]

**Supplemental Table I.** Correlations between final accuracy and retention rates

| Exp 1 |  | Short delay | | Long delay | |
| --- | --- | --- | --- | --- | --- |
|  |  | Unhindered | Hindered | Unhindered | Hindered |
|  | Abstract | 0.76*** | 0.73*** | 0.71*** | 0.76*** |
|  | Concrete | 0.79*** | 0.70*** | 0.79*** | 0.64*** |
| Exp 2 |  |  |  |  |  |
|  | Abstract | 0.63*** | 0.61*** | 0.60*** | 0.44** |
|  | Concrete | 0.71*** | 0.76*** | 0.75*** | 0.80*** |
| *Note.* Final accuracy was operationalized as the random intercept obtained from a multilevel regression analysis per stimulus type and verbalization condition combination (i.e., four in total) with backward-coded trial as independent variable; retention rates were operationalized as the proportion of choices for the stimulus with the highest expected value; *** *p* < 0. 001, ** *p* < 0.01 | | | | | |
