# Supplementary material for: What’s in a name: The role of verbalization in reinforcement learning
Source: Psychon Bull Rev. 2024 May 20;31(6):2746–57. doi: 10.3758/s13423-024-02506-3 (PMC11680654; doi:10.3758/s13423-024-02506-3)
Supplement: Supplementary file 4 — Supplementary file4 (DOCX 23.2 KB) [file 13423_2024_2506_MOESM4_ESM.docx]

**Supplemental Table II.** Comparison between estimates from multilevel logistic regression analysis on accuracy when removing timed-out responses versus when treating these timed-out responses as incorrect responses

|  | Effect | Estimate without timed-out responses | | Estimate with timed-out responses treated as incorrect responses | |
| --- | --- | --- | --- | --- | --- |
| Exp 1 | Intercept | 0.84 | *** | 0.83 | *** |
|  | Stim type | 0.24 | *** | 0.24 | *** |
|  | Verb cond | 0.05 |  | 0.05 |  |
|  | Trial | 1.55 | *** | 1.55 | *** |
|  | Stim type x verb cond | 0.01 |  | 0.01 |  |
|  | Stim type x trial | 0.26 | ** | 0.25 | ** |
|  | Verb cond x trial | -0.05 |  | -0.08 |  |
|  | Stim type x verb cond x trial | -0.03 |  | -0.01 |  |
| Exp 2 | Intercept | 0.71 | *** | 0.57 | *** |
|  | Stim type | 0.24 | *** | 0.24 | *** |
|  | Verb cond | 0.15 | ** | 0.13 | ** |
|  | Trial | 1.24 | *** | 1.19 | *** |
|  | Stim type x verb cond | 0.06 | ** | 0.05 | ** |
|  | Stim type x trial | 0.37 | *** | 0.35 | *** |
|  | Verb cond x trial | 0.23 | ** | 0.15 |  |
|  | Stim type x verb cond x trial | 0.00 |  | -0.02 |  |
| *Note.* Stim type = stimulus type; verb cond = verbalization condition; *** indicates  *p*-values < .001 ; ** *p*-values < .01 | | | | | |
