# Supplementary material for: What’s in a name: The role of verbalization in reinforcement learning
Source: Psychon Bull Rev. 2024 May 20;31(6):2746–57. doi: 10.3758/s13423-024-02506-3 (PMC11680654; doi:10.3758/s13423-024-02506-3)
Supplement: Supplementary file 5 — Supplementary file5 (DOCX 21.7 KB) [file 13423_2024_2506_MOESM5_ESM.docx]

**Supplemental Table III.** Number of participants (out of 50) that achieved above-chance level performance at the final eight trials of the task

|  |  | Unhindered | Hindered |
| --- | --- | --- | --- |
| Exp 1 | Abstract | 35 | 37 |
|  | Concrete | 41 | 41 |
| Exp 2 | Abstract | 32 | 30 |
|  | Concrete | 38 | 31 |
