# Supplementary material for: What’s in a name: The role of verbalization in reinforcement learning
Source: Psychon Bull Rev. 2024 May 20;31(6):2746–57. doi: 10.3758/s13423-024-02506-3 (PMC11680654; doi:10.3758/s13423-024-02506-3)

**Supplemental Figure I.** *Time it took participants to come up with a name for the abstract (red) and concrete (blue) stimuli in ascending order. Error bars indicate one standard error of the mean. Grey error bars indicate stimuli that have been removed based on the pilot results*


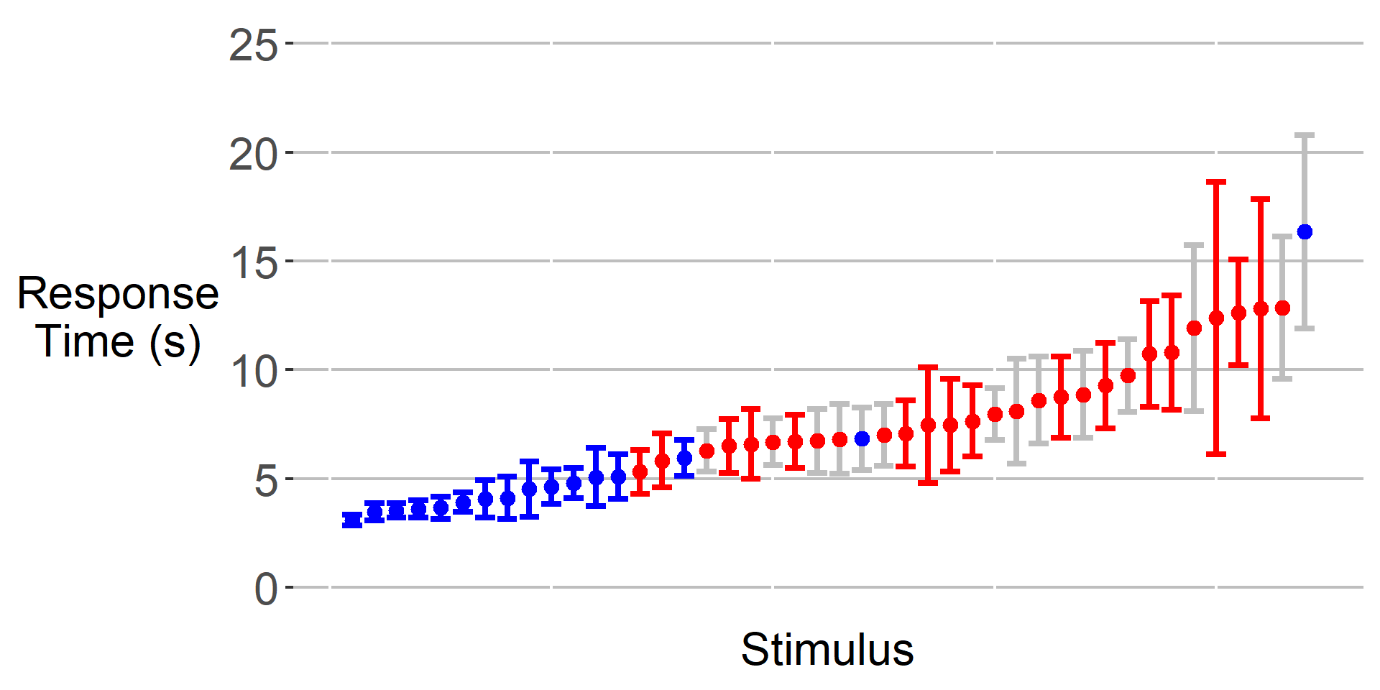

Supplement: Supplementary file 6 — Supplementary file6 (DOCX 140 KB) [file 13423_2024_2506_MOESM6_ESM.docx]
