# Supplementary material for: What’s in a name: The role of verbalization in reinforcement learning
Source: Psychon Bull Rev. 2024 May 20;31(6):2746–57. doi: 10.3758/s13423-024-02506-3 (PMC11680654; doi:10.3758/s13423-024-02506-3)

**Supplemental Figure II.** *Self-reported difficulty in naming the abstract (red) and concrete (blue) stimuli. Error bars indicate one standard error of the mean. Grey error bars indicate stimuli that have been removed based on the pilot results*


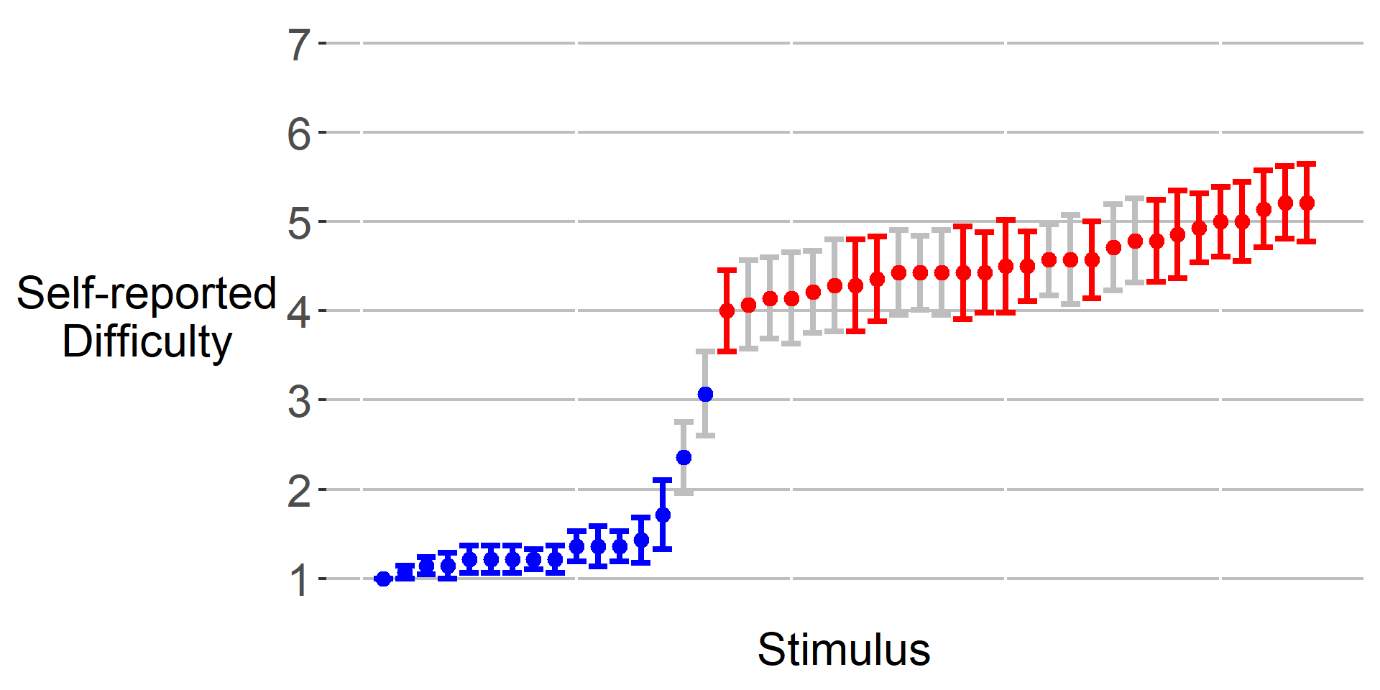

Supplement: Supplementary file 7 — Supplementary file7 (DOCX 136 KB) [file 13423_2024_2506_MOESM7_ESM.docx]
