# Supplementary material for: What’s in a name: The role of verbalization in reinforcement learning
Source: Psychon Bull Rev. 2024 May 20;31(6):2746–57. doi: 10.3758/s13423-024-02506-3 (PMC11680654; doi:10.3758/s13423-024-02506-3)

**Supplemental Figure III.** *Self-reported similarity between the two stimuli in the abstract (red) and concrete (blue) pairs. Error bars indicate one standard error of the mean. Grey error bars indicate pairs from which at least one of the stimuli has been removed based on the pilot results.*


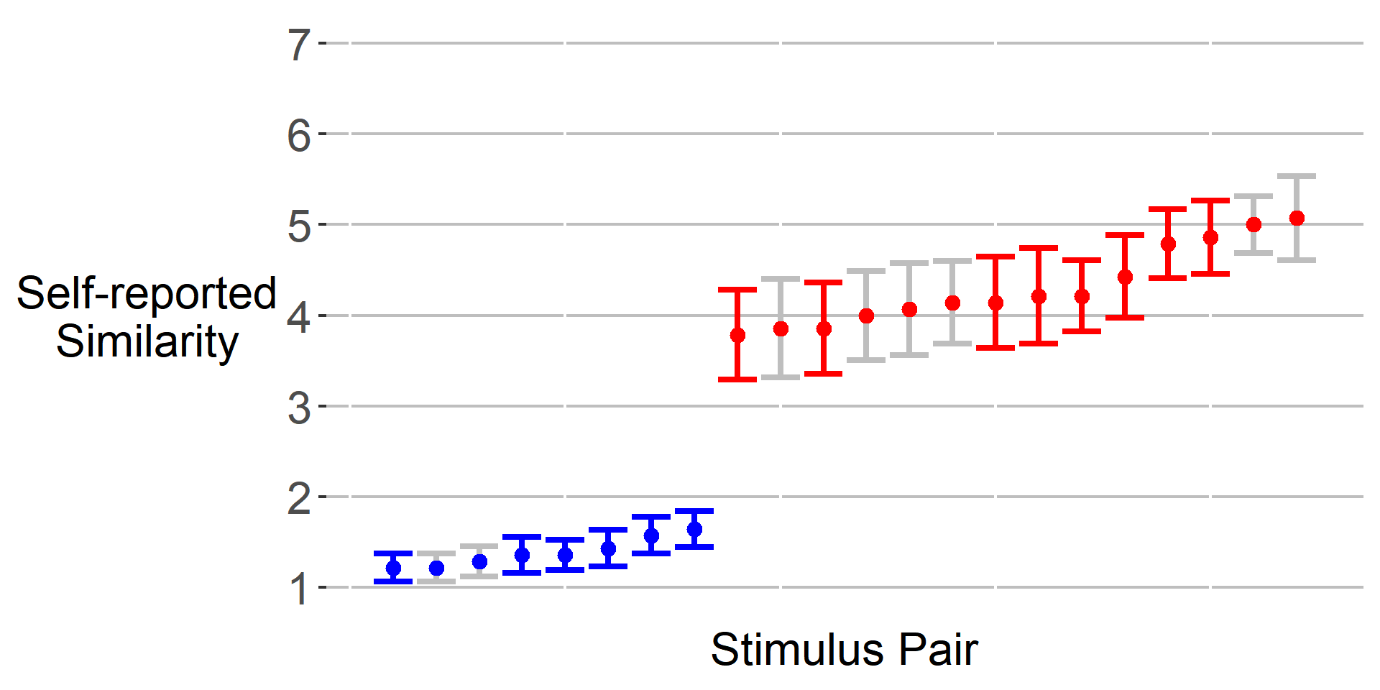

Supplement: Supplementary file 8 — Supplementary file8 (DOCX 55.3 KB) [file 13423_2024_2506_MOESM8_ESM.docx]
